# Possible cannabinoid-induced lactic acidosis requiring emergent dialysis

**DOI:** 10.1177/2050313X241265069

**Published:** 2024-07-26

**Authors:** Marcus H Cummins, Brandon J Croft

**Affiliations:** 1 Department of Internal Medicine, University of California San Francisco—Fresno, Fresno, CA, USA; 2 Department of Pulmonary and Critical Care Medicine, University of California San Francisco—Fresno, Fresno, CA, USA

**Keywords:** Lactic acidosis, type B lactic acidosis, cannabinoids, marijuana

## Abstract

Lactic acidosis is a common finding in the intensive care unit and is associated with increased mortality. We present the case of a 42-year-old male with alcohol use disorder and cirrhosis who developed sudden onset shortness of breath while smoking marijuana. He was found to have a lactic acid level of 25.6 mmol/L with a significant anion gap metabolic acidosis requiring emergent dialysis. He was hypertensive without evidence of tissue hypoperfusion. His profound type B lactic acidosis was primarily attributed to a rare manifestation of cannabinoid toxicity. At a clinic visit 3 months later, he was doing well and had not smoked marijuana since his discharge.

## Introduction

Lactic acidosis is a common finding in patients admitted to the intensive care unit (ICU) and is associated with increased mortality.^1^ It is usually caused by decreased oxygen delivery and tissue hypoxia, or type A lactic acidosis. Type B lactic acidosis is defined as hyperlactatemia in the absence of tissue hypoxia. There are several potential etiologies, including medications, thiamine deficiency, malignancy, diabetes, liver disease, and sepsis.^2–4^ In this report, we describe the case of a young male found to have severe type B lactic acidosis that was primarily attributed to a rare manifestation of cannabinoid toxicity.

## Case report

A 42-year-old male with alcohol use disorder and cirrhosis presented with acute-onset shortness of breath and was hypertensive, tachycardic, and tachypneic. He was saturating 100% on room air, but had accessory muscle use. Arterial blood gas revealed pH 7.18 (ref 7.35–7.45), pCO_2_ 10 mmHg (ref 35-45), and HCO_3_ of 4 mmol/L (ref 21-28). Serum chemistry panel revealed Na 142 mmol/L (ref 135–145), K 4.1 mmol/L (ref 3.5–5.3), Cl 112 mmol/L (ref 98–110), and bicarbonate 5 mmol/L (ref 22–28). The reported anion gap was 25 mmol/L (ref 8–16), but his albumin-corrected anion gap was 28.3 mmol/L as his serum albumin was 2.7 g/dL (ref 3.4–4.8). Serum lactate was 25.6 mmol/L (ref 0.5–2.2), B-Hydroxybutyrate was 4.6 mg/dL (ref 0.2–2.8), and blood alcohol level was 0.018 g/dL (ref ⩽ 0.010). Urine drug screen was positive for cannabinoids. Methanol, ethylene glycol, and salicylate levels were negative. The osmolal gap was 20.8 mOsm/kg. The toxicology team stated that the presence of an osmolal gap and anion gap without hypocalcemia (calcium 8.7 mg/dL (ref 8.5–10.5)) made toxic alcohol ingestion less likely. Kidney function was normal and urine calcium oxalate crystals were not tested for. Given the severe acidosis, nephrology recommended emergent dialysis. Prior to dialysis, the patient became altered and agitated and was intubated for airway protection. He was started on a sodium bicarbonate drip, a temporary dialysis catheter was placed, and he was admitted to the ICU.

He received one session of hemodialysis over the first night of admission. Repeat lactic acid was 13.0 mmol/L (ref 0.5–2.2). Later that evening, it was 3.7 mmol/L (ref 0.5–2.2) and 6 hours later it normalized at 2.1 mmol/L (ref 0.5–2.2). In addition, he was started on ceftriaxone and metronidazole for imaging findings consistent with pancolitis and empiric high-dose thiamine given his history of alcohol use disorder.

He was extubated on ICU day three. The following morning, he was asked to recall the events leading up to his hospitalization. He remembered everything and did not recall ingesting anything out of the ordinary. He did, however, report smoking significantly more marijuana than usual. He purchased his marijuana from the street and could not confirm exactly what was in it. He said he usually took a “hit” every 5-to-6 hours, but that day he was smoking continuously until he became so short of breath that he had to call 911.

He was downgraded from the ICU and discharged from the hospital 7 days later. At a clinic visit 3 months later, he was doing well and had not smoked marijuana since his discharge.

## Discussion

The underlying pathophysiology of all causes of type A lactic acidosis is tissue hypoxia. Common etiologies include shock, severe hypoxemia, carbon monoxide poisoning, and severe anemia.^2^ Type B lactic acidosis can be categorized based on the underlying pathophysiology. Metformin, nucleoside reverse-transcriptase inhibitors, alcohol (toxic alcohols, methanol, ethylene glycol, diethylene glycol), salicylates, cyanide, and propofol interfere with oxidative phosphorylation.^2^ Antibiotics, particularly linezolid, may interfere with mitochondrial adenosine triphosphate synthesis.^3^ Beta-2 agonists, such as albuterol, stimulate aerobic glycolysis.^4^ Thiamine deficiency impairs pyruvate dehydrogenase activity. Solid malignancies have been associated with increased glycolytic tumor activity and tumor tissue hypoxia. Diabetes mellitus has been associated with type B lactic acidosis, although the underlying mechanism is not well understood. Finally, liver disease and sepsis both result in decreased clearance of lactic acid.^2^

Our patient had a severe anion gap metabolic acidosis. Lactic acid accounted for the majority of the anion gap. His B-Hydroxybutyrate and blood alcohol levels were mildly elevated so there was also likely a component of alcoholic ketoacidosis. Other causes of high anion gap metabolic acidosis (ethylene glycol poisoning, methanol intoxication, salicylate poisoning, and renal failure) were excluded. He also had a respiratory alkalosis (expected pCO2 using Winter’s formula was 12–16). This was likely a compensatory response to his profound metabolic acidosis. The severity of his acidosis necessitated emergent dialysis. There are no clear guidelines on the indications for renal replacement therapy, but severe acidosis (commonly defined as pH ⩽ 7.2) is a commonly accepted indication.^5^ Cases of severe type B lactic acidosis due to lymphoma and metformin that required dialysis have also been reported.^6,7^

Our patient had multiple potential etiologies contributing to type B lactic acidosis. He received empiric high-dose thiamine. A serum thiamine level was not sent, but he did not have any features of severe thiamine deficiency.^8^ Sepsis was a consideration, as he was tachycardic and tachypneic and had a potential source of infection identified on abdominal imaging. He received empiric antibiotics but did not have a fever or leukocytosis. His tachycardia can be explained by cannabinoid toxicity, as can agitation and altered mentation. His tachypnea was likely a compensatory response as mentioned above.^9^ He had cirrhosis (Meld-Na 22, Child Class C) with evidence of hepatic synthetic dysfunction which impaired his ability to clear lactic acid efficiently. Finally, the presence of an osmolal gap raised concern for toxic alcohol ingestion. However, the history was negative for toxic alcohol ingestion and lactic acidosis alone has been associated with an elevated osmolal gap.^10,11^ There are three proposed mechanisms for this association. Certain medications administered to patients in the ICU, such as lorazepam, multivitamin preparations, and nitroglycerin, are stored in propylene glycol, which can cause both an elevated osmolal gap and lactic acidosis. In patients with alcohol use disorder, endogenous compounds, such as glycerol, acetone, and acetone metabolites can elevate the osmolal gap. If these patients have concurrent hepatic dysfunction, type 2 lactic acidosis can develop due to decreased clearance. Finally, cell breakdown during lactic acidosis is thought to lead to the release of other products of glycogen breakdown that can elevate the osmolal gap.^12^ The latter two are suspected mechanisms in our patient.

The only notable feature of the history was significant marijuana use. There have been a few reports of marijuana-associated lactic acidosis. A 69-year-old female with daily marijuana use presented with shortness of breath and abdominal pain. Her lactic acid level was 8.8 mmol/L and rose to 13.6 mmol/L. There was no evidence of decreased oxygen delivery and the lactic acidosis resolved without treatment.^13^ An 18-year-old male found unconscious with rapid, shallow breathing requiring intubation had a lactic acid level of 10.6 mmol/L. After extubation, the history was only significant for synthetic marijuana use.^14^ Finally, a 57-year-old female with frequent cannabis use presented with gastrointestinal symptoms and a lactic acid level of 4.8 mmol/L; however, thiamine deficiency was also suspected.^15^ Unfortunately, the underlying mechanism for lactic acidosis due to marijuana is unknown.

## Conclusion

In light of this and the history our patient provided, his profound lactic acidosis was primarily attributed to a rare manifestation of cannabinoid toxicity. This was exacerbated by impaired hepatic clearance of lactic acid, thus requiring emergent dialysis. Sepsis and thiamine deficiency may have also contributed, but neither alone, nor in combination, are consistent with this degree of lactic acidosis.
